# Analysis of the Prevalence and Factors Associated with Nocturia in Adult Korean Men

**DOI:** 10.1038/srep41714

**Published:** 2017-01-31

**Authors:** So Young Kim, Woojin Bang, Min-Su Kim, Bumjung Park, Jin-Hwan Kim, Hyo Geun Choi

**Affiliations:** 1 Department of Otorhinolaryngology-Head & Neck Surgery and Cancer Research Institute, Seoul National University College of Medicine, Seoul, Korea; 2 Department of Urology, Hallym University Sacred Heart Hospital, Hallym University Sacred Heart Hospital, Anyang, Korea; 3 Department of Otorhinolaryngology-Head & Neck Surgery, Korea University Ansan Hospital, Ansan, Korea; 4 Department of Otorhinolaryngology-Head & Neck Surgery, Hallym University College of Medicine, Anyang, Korea; 5 Department of Otorhinolaryngology-Head & Neck Surgery, Hallym University College of Medicine, Seoul, Korea

## Abstract

This study investigated the prevalence of and factors associated with nocturia in Korean men. A total of 92,626 participants aged between 19 and 103 years from the 2011 Korean Community Health Survey (KCHS) were enrolled. Simple and multiple logistic regression analyses with complex sampling investigated participants’ personal health and socioeconomic and disease factors. The prevalence of nocturia ≥1 time and ≥2 times/night was 41.8% and 17.6%, respectively, and nocturia increased with age (1.44 [1.39–1.50] for each 10-year increase, P < 0.001). Lower income levels (lowest, 1.27 [1.19–1.36]; low-middle, 1.13 [1.07–1.19]; upper-middle, 1.00 [0.95–1.06], P = 0.022) and higher levels of stress (severe, 1.38 [1.23–1.55]; moderate, 1.23 [1.16–1.31]; some, 1.11 [1.05–1.16]) exhibited dose-dependent relationships with nocturia (≥1 time; P < 0.001). Low education level (1.27 [1.20–1.36]), long sleep duration (1.33 [1.18–1.50]), and type of occupation showed significant associations with nocturia (≥1 time; P < 0.001). Underweight (1.19 [1.05–1.34]), hypertension (1.09 [1.03–1.15]), diabetes mellitus (1.32 [1.23–1.41]), hyperlipidaemia (1.28 [1.20–1.35]), and cerebral stroke (1.63 [1.40–1.89]) were significantly related to nocturia (≥1 time; P < 0.001). Married men were less likely to experience nocturia ≥2 times per night (0.72 [0.64–0.82], P < 0.001).

Lower urinary tract symptoms (LUTS) are irritating symptoms that are present in approximately 41% of adult men^1^. Nocturia is the most common (accounting for up to 69% of LUTS) and most persistent LUTS in men^2^,^3^,^4^,^5^,^6^. Nocturia has detrimental effects on quality of life, impairs work productivity, and increases mortality by elevating the risk of fractures, especially in the elderly^2^,^7^. Due to the intractable nature of nocturia and the recent ageing of the population, the economic burden of nocturia is enormous and continues to increase. Thus, a significant amount of attention has been devoted to evaluating the prevalence and associated risk factors of nocturia.

The prevalence of nocturia has been estimated to range from 37% to 69%^3^,^4^,^5^,^6^. Recent figures based on meta-data estimate an annual incidence of nocturia of 0.4% for young adults and 11.5% for the elderly^6^,^7^. This heterogeneity can be explained by differences in study populations, survey protocols, and definitions of nocturia. To standardize and improve the consistency in nocturia research, the International Continence Society (ICS) has defined nocturia as a complaint of 1 or more episodes of waking up to void per night^8^,^9^.

The underlying pathophysiologies of nocturia are complex and involve interactions between several anatomic, neurologic, and metabolic factors^10^. Benign prostatic obstruction (BPO) and overactive bladder (OAB) are two main causes of nocturia and other LUTS in adult men^11^. Multiple conditions related to BPO and OAB can underlie nocturia. Additionally, metabolic and neurologic factors are related to LUTS^10^, and salt-water imbalances and nocturnal polyuria can also result in nocturia^12^. Indeed, obesity, cardiovascular disease, diabetes mellitus, sleep disorders, psychiatric problems and behavioural and environmental factors are suggested to be related to nocturia^13^,^14^. Thus, comprehensive adjustments for these factors are warranted to prevent biases from confounding effects to better elucidate the factors related to nocturia.

Although numerous previous studies have examined the prevalence of nocturia, there are no studies that include large populations with wide age ranges. The purpose of the present study is therefore to explore the prevalence of and factors related to nocturia in Korean men. The Korean Community Health Survey (KCHS) is a large, nationwide, stratified sampled investigation representative of the Korean population. The KCHS encompasses a variety of aspects including medical, demographic, socioeconomic, behavioural and psychological factors, thus enabling an extensive consideration of these variables in terms of their relationships with nocturia.

## Results

**Figure 1:**
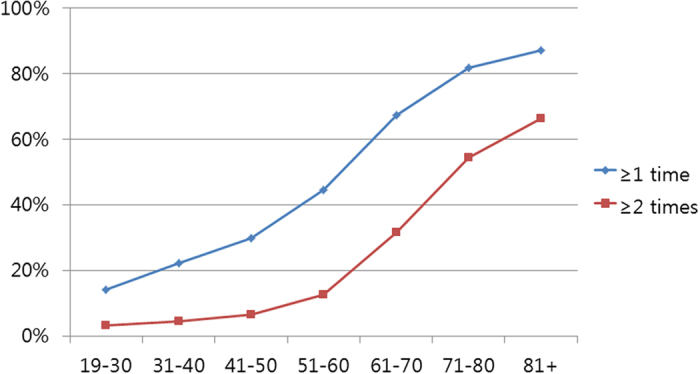
The prevalence of nocturia according to age group.

Among the participants, 41.8% and 17.6% reported having nocturia ≥1 time and ≥2 times per night, respectively (Table 1). The prevalence of nocturia increased with age (Fig. 1), with the prevalence steeply increasing after the age of 51 years. The mean ages of the groups with nocturia ≥1 time and ≥2 times per night were 53.0 and 59.7 years old, respectively, and these ages were significantly higher than those of the control group (P < 0.001). Marital status, level of education, type of occupation, level of income, body mass index (BMI), smoking status, alcohol consumption, sleep duration, level of stress, and medical history of hypertension, diabetes mellitus, hyperlipidaemia, and cerebral stroke differed significantly between the nocturia (≥1 time and ≥2 times per night) and control groups (all Ps < 0.001; Table 1).

For each 10-year increase in age, the prevalence of nocturia (≥1 time) increased 1.44-fold according to the multiple regression analysis (95% CI = 1.39–1.50, P < 0.001; Table 2). Underweight was also significantly associated with nocturia (≥1 time; AOR = 1.19, 95% CI = 1.05–1.34, P = 0.022), while obesity did not exhibit a significant relationship with nocturia (≥1 time). A medical history of hypertension, diabetes mellitus, hyperlipidaemia, and cerebral stroke was associated with a 1.09−(95% CI = 1.03–1.15), 1.32−(95% CI = 1.23–1.41), 1.28−(95% CI = 1.20–1.35), and 1.63−(95% CI = 1.40–1.89) fold increase in the prevalence of nocturia (≥1 time), respectively (all Ps < 0.001; Table 2). Alcohol consumption showed a dose-dependent association with nocturia (≥1 time) (AOR [95% CI] = 1.08 [1.01–1.15] < 1.10 [1.03–1.16] < 1.15 [1.09–1.21] for consuming alcohol ≤1 time per month, 2–4 times per month, and ≥2 times per week, respectively, P < 0.001). Stress level also exhibited a dose-dependent relationship with nocturia (AOR = 1.11 < 1.23 < 1.38 for some, moderate, and severe stress, respectively, P < 0.001; Table 2). Sleep duration exhibited a dose-dependent relationship with nocturia (≥1 time; AOR [95% CI] = 0.91 [0.88–0.95] < 1 < 1.33 [1.18–1.50] for ≤6 h, 7–8 h and ≥9 h, respectively, P < 0.001).

Lower education level was significantly related to nocturia (≥1 time) in a dose-dependent manner (AOR = 1.27 [1.20–1.36] > 1.04 [0.99–1.09] for low >middle levels; both Ps < 0.001). A lower income level was significantly and dose-dependently associated with increased nocturia (≥1 time) (AOR [95% CI] = 1.27 [1.19–1.36] > 1.13 [1.07–1.19] for the lowest >low-middle levels, P < 0.001). In addition, the participant’s type of occupation showed a significant correlation with nocturia. The group including production workers, engineers, farmers, fishers, labourers, and soldiers (AOR = 1.18, 95% CI = 1.11–1.26, P < 0.001) and the unemployed and student group (AOR = 1.21, 95% CI = 1.13–1.29, P < 0.001) maintained significant correlations with nocturia (≥1 time). Married men did not show a significant association with nocturia (≥1 time) in the multiple logistic regression analysis.

Most of the relationships between nocturia and the above-mentioned variables were consistent when analysed for the group with nocturia ≥2 times per night (Table 2). Additionally, married men were significantly less likely to exhibit nocturia (AOR = 0.72, 95% CI = 0.64–0.82, P < 0.001). In contrast, alcohol consumption was not significantly related to nocturia (≥2 times).

## Discussion

Among adult Korean men, 41.8% and 17.6% reported having nocturia 1 or more times and 2 or more times per night, respectively. These figures are somewhat higher than those reported in several former studies^5^ but lower than those reported the prevalence in a US population study (69% for ≥1 and 28% for ≥2 times) that used the same definition of nocturia as the present study^6^. These discrepancies could partly originate from the low response rate in the previous study^6^ of approximately 59% (compared to 90.0% in the present study) and the older population (40% or more above the age of 40 years old vs. ≥19 years old in the present study) that it included.

Consistent with previous studies, the prevalence of nocturia increased with age^6^,^15^. Nocturia can result from a small bladder capacity, nocturnal polyuria, or 24 h polyuria or can have a mixed aetiology^6^,^16^. Degenerative changes such as prostate problems^6^ and diminished bladder compliance and deregulation of nocturnal antidiuretic hormone are plausible mechanisms of nocturia in the elderly^16^. Nocturnal polyuria also increases with age^17^. Nocturia in the elderly has been associated with a high prevalence of chronic medical diseases and related medications^6^, as well as with increases in sleep disorders, depression and frequency of waking up at night^18^.

Underweight has been significantly correlated with nocturia^19^. In contrast to our results, many previous studies have suggested that obesity is associated with nocturia^20^. However, a recent study reported that underweight was correlated with increased mortality in nocturia patients^21^. Underweight subjects may have decreased nutritional reserves and weakened immunity and may therefore be more susceptible to infection^22^,^23^,^24^. Moreover, the increased comorbidity in the elderly may result in underweight, and these chronic diseases could be associated with nocturia. The inverse relationships, i.e., nocturia resulting in underweight due to a combination of LUTS, insufficient sleep quality, and consequent medical complications, are also possible. Because there was only a small proportion of obese subjects in our Korean study population, obesity-related complications might not have been detected.

Medically compromised subjects with hypertension, diabetes mellitus, hyperlipidaemia, and cerebral stroke were more likely to exhibit nocturia with statistical significance. Indeed, nocturia is known to be associated with numerous medical disorders, including metabolic syndrome and cardiovascular disease^25^. Importantly, diabetes mellitus and hypertension are related to persistent nocturia^2^, and diabetes mellitus is associated with osmotic polyuria^6^. In addition, the microvascular complications of diabetic nephropathy may lead to polyuria, and diabetic neuropathy may cause impaired bladder sensation and increased residual urine mediated by a deregulated secretion of vasopressin^26^,^27^. In hypertensive patients, high blood pressure may affect glomerular filtration, tubular transport, and other renal functions associated with nocturia^28^. Furthermore, hypertension has been suggested to be related to defects in the nitric-oxide pathway that alter pressure-natriuresis and sodium retention and cause compensatory nocturnal polyuria^29^. Additionally, central haemodynamic parameters, such as central blood pressure, cardiac output, pulse wave velocity and vascular stiffness, have been suggested to be associated with nocturia^30^.

In this study, stress was related with nocturia in a dose-dependent manner. LUTS are controlled by the central and peripheral nervous systems, and disruptions in these systems due to stress are among the pathophysiologic mechanisms of overactive bladder^31^,^32^. Nocturia can also elevate stress levels, as sleep disturbances due to nocturia may lead to daytime fatigue and psychiatric disorders^25^. In our study, longer sleep times were related to nocturia. In simple terms, longer sleep times may be associated with a higher likelihood of waking up and voiding. Moreover, excessive sleep duration might interrupt circadian rhythms, which regulate urine output via diuretic and anti-diuretic hormones and other clock gene expressions^33^. On the other hands, nocturia could cause sleep disturbances and thereby prolong the total required sleep duration^33^.

Several socioeconomic variables exhibited associations with nocturia with statistical significance in this study. Low education and income levels were associated with nocturia. More highly educated persons generally have a better knowledge of and focus greater attention on their health^34^,^35^. In contrast, subjects with a lower socioeconomic status find access to healthcare more challenging and are more likely to exhibit poor hygiene.

The prevalence of nocturia differed according to the participants’ type of occupation with statistical significance. First, the unemployed or student group exhibited the highest rates of nocturia, at 1.21- (≥1 time) and 1.44-fold (≥2 times) greater rates than those of the management, expert, specialist, and clerk group. Stressful conditions due to preparing for examinations and the prolonged sedentary and unhealthy lifestyles associated with being unemployed or a student may have contributed to the increased nocturia prevalence in this group. Indeed, studies have reported that compared with unemployed and/or part-time workers, full-time employees exhibit lower levels of stress and depression, healthier and less unhealthy eating, more physical activity, and lower levels of smoking and drinking^36^. Regarding the inverse relationship, conditions that are physically comorbid with nocturia may lead to unemployment. Second, farmers, fishers, labourers, and soldiers exhibited 1.18- (≥1 time) and 1.21-fold (≥2 times) elevations in the prevalence of nocturia compared with the manager, expert, specialist, and clerk group. The repeated retention of urine and heavy pressure on the pelvic floor, which are expected to be more common in physical workers, could induce bladder dysfunction^37^. Disturbed circadian rhythms among labourers due to work shifts, particularly those at night, have been suggested to increase nocturia mainly due to a decreased nocturnal bladder capacity^33^. Additionally, cold exposure during the daytime among individuals who work outdoors may induce detrusor overactivity and thereby result in nocturia^38^.

Marriage was inversely associated with nocturia (≥2 times). Married men exhibited a increased prevalence of nocturia in the univariate analysis, and this relationship could be explained by several confounders including the higher age of married compared with unmarried men. Additionally, the more active sexual behaviours and elevated sex hormones in married persons may be beneficial for LUT function. Sex hormones have been suggested to be associated with better LUT function, and the frequent nocturia (>3 times compared to ≤3 times) may reduce sex hormone levels^39^,^40^.

The effects of smoking and alcohol were inconsistent in the present study. In contrast to common knowledge, current smokers exhibited a lower prevalence of nocturia than non-smokers. Similarly to the present results, a recent study also demonstrated the lower prevalence of nocturia compared to that of former smoker with the probable links of nicotine effects of increasing arginine vasopressin secretion and detrusor contractile response^41^. In addition, this finding might be due to the health-seeking behaviours (quitting smoking) of older or medically compromised subjects. Alcohol consumption exhibited a dose-dependent relationship with nocturia (≥1 time). As the simple logistic regression analysis demonstrated an inverse dose-dependent relationship of nocturia with alcohol consumption, adjusting for possible confounders is critical to identifying the true associations of alcohol consumption with nocturia.

The present results are subject to limitations related to several factors. Due to the cross-sectional study design, the causality of the relationships between the variables and nocturia could not be determined. Additionally, the self-reported questionnaires introduced potential recall bias. The frequency-volume chart is a standardized diagnostic method for nocturia. Although the nocturia using questionnaire survey was associated with the frequency-volume chart data, the comorbid conditions were suggested to be different^42^. It was reported that nocturia using questionnaire survey could be exaggerate the prevalence or incidence of nocturia^43^. Thus, the present data should be interpreted with caution and compared with studies with comparable study designs (Supplementary note S2). Moreover, the possibility that other unconsidered variables affected nocturia cannot be excluded. Finally, because this study was based on Korean adult men, the application of the identified relationships between the different variables and nocturia in other groups may be limited. However, our study strengths overcome its limitations. First, we considered numerous variables that included demographic, socioeconomic, medical and psychological factors. In addition, our homogenous ethnic population enabled us to minimize the confounding effects of heterogeneous ethnic backgrounds and to assess gender-specific characteristics of nocturia. Nocturia was defined and surveyed according to the standard ICS guidelines. Furthermore, we performed two analyses for groups with nocturia ≥1 time and ≥2 times per night. This approach was based on several recent studies that indicated that nocturia ≥1 time per night is not bothersome, whereas ≥2 voids per night represent clinically meaningful nocturia^44^,^45^. Most importantly, a very large study population was selected using stratified weighted sampling methods to represent the Korean population, thus strengthening the statistical power of the present study. Future studies with prospective and preclinical designs will be helpful for determining the causal relations between the various factors presently identified and nocturia.

## Methods

### Study Population and Data Collection

This study was approved by the Institutional Review Board of the Korea Centers for Disease Control and Prevention (IRB No. 2011-05CON-04-C). Written informed consent was obtained from each participant prior to the survey. All KCHS procedures are conducted in accordance with the guidelines and regulations provided by the Korea Centers for Disease Control and Prevention.

This study used a cross-sectional design that employed data from the KCHS. Data from the 2011 KCHS were analysed. The data were collected by the Korean Centers for Disease Control and Prevention. The survey gathered information through face-to-face, paper-assisted personal interviews between trained interviewers and respondents. The sample size for the KCHS was 900 subjects in each of the 253 community units, including 16 metropolitan cities and provinces. The KCHS used a two-stage sampling process. The first stage selected the sample area (district/street/village) as the primary sample unit according to the number of households in the area using a probability proportional to size sampling method. In the second stage, the number of households in the selected district/street/village sample was identified to create a household directory. Sample households were selected using systematic sampling methods. This process was applied to ensure that the sample units were representative of the entire population^46^. To enable statistical representation of the population, the data collected from the survey were weighted by statisticians based on the sample design^47^.

**Figure 2:**
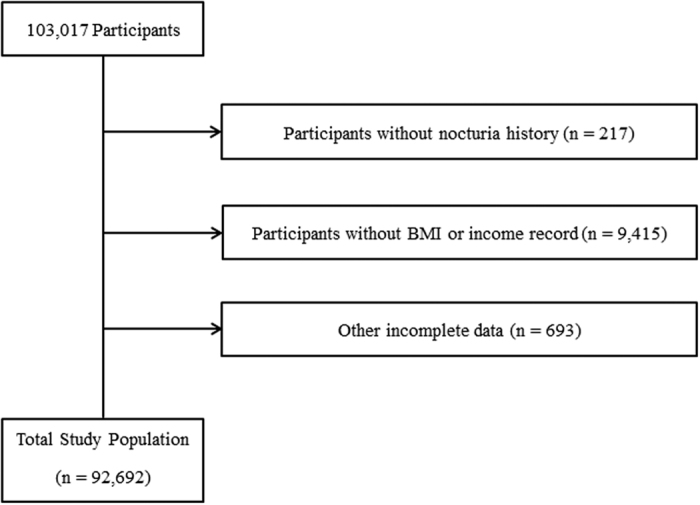
A schematic illustration of the participant selection for the present study. Of the 103,017 total participants, participants who did not have a history of nocturia (n = 217), were missing BMI or income records (9,415) and had other incomplete data (693) were excluded. The data for the 92,692 participants with complete data were analysed.

Of the 103,017 total male participants aged from 19 to 103 years old, we excluded the following participants from this study: those who did not complete the nocturia survey (217 participants); those without recorded height, weight, or income data (9,415 participants); and those who had incomplete data regarding marital status, education level, occupation, smoking, alcohol consumption history, sleep hours, stress level, and history of hypertension, diabetes mellitus, hyperlipidaemia, or cerebral stroke (558 participants). Ultimately, 92,692 participants were included in this study (Fig. 2).

### Survey

Marital status, including common-law marriages, was assessed. To measure physical activity, the participants were asked about the number of days in the previous week that they had spent more than 10 minutes walking. To explore the influence of educational level, uneducated participants and those who had graduated only from elementary or middle school were assigned to the “low” education group. High school education comprised the “middle” education group, and junior college, graduate school and college graduates formed the “high” education group. Occupation was classified into the following 5 groups according to physical activity: manager, expert, specialist, and clerk; service worker and salesperson; technician, mechanic, production worker, and engineer; farmer, fisher, labourer, and soldier; and unemployed and student. Participants under 110 cm or 30 kg were excluded from this study. Using the criteria for the Asia-Pacific region^48^, three body mass index (BMI, kg/m^2^) groups were created: low BMI, <18.5 kg/m^2^; normal BMI, 18.5–25 kg/m^2^; and high BMI, ≥25 kg/m^2^ 49. Using the methods recommended by the Organization for Economic Cooperation and Development^50^ (i.e., dividing household income by the square root of the number of household members), monthly income was divided into the lowest, low-middle, upper-middle, and highest quartiles. Smoking status was divided into 3 groups: non-smoker, past smoker, and current smoker. Past smokers who had quit smoking less than 1 year prior to the survey were included in the current smoker group. Alcohol consumption was divided into the following three categories: none, ≤1 time a month, 2–4 times a month, and ≥2 times a week. The amount of sleep reported was divided into three groups: ≤6 h per day, 7–8 h per day, and ≥9 h per day. Participants who slept less than 3 hours per night were excluded from this study. The participants were asked if they usually felt stress, and their stress levels were divided into the following four groups: no stress, some stress, moderate stress, and severe stress.

The participants were asked about their history of other comorbidities, such as hypertension, diabetes mellitus, hyperlipidaemia, and cerebral stroke, and those who reported a history of any of these diseases as diagnosed by a medical doctor were recorded as positive.

To determine nocturia, the participants were asked the question, “How many times did you typically get up at night to urinate in the past month?” The current ICS definition of nocturia (≥1 void per night) was used^8^, and a secondary analysis was performed for those with ≥2 voids per night.

### Statistical Analysis

The differences in mean age and walking days between the normal participants (control) and nocturia participants were compared using linear regression analysis with complex sampling. The differences in the rates of marriage, education level, occupation, income level, BMI, smoking, alcohol consumption history, sleep hours, stress level, and history of hypertension, diabetes mellitus, hyperlipidaemia, and cerebral stroke were compared using chi-squared tests with Rao-Scott correction (Table 1).

To identify the associations between the related factors and nocturia (≥1 time and ≥2 times), simple and multiple logistic regression analyses with complex sampling were used (Supplementary Table S1 and Table 2). Two-tailed analyses were conducted, and P-values below 0.05 were considered significant. The adjusted odds ratio (AOR) and 95% confidence intervals (CIs) for nocturia were calculated. All results are presented as weighted values. The results were analysed using SPSS ver. 21.0 (IBM, Armonk, NY, USA).

## Additional Information

**How to cite this article**: Kim, S. Y. *et al*. Analysis of the Prevalence and Factors Associated with Nocturia in Adult Korean Men. *Sci. Rep.* **7**, 41714; doi: 10.1038/srep41714 (2017).

**Publisher's note:** Springer Nature remains neutral with regard to jurisdictional claims in published maps and institutional affiliations.

## Supplementary Material

Supplementary Table S1

Supplementary Notes S2

## Figures and Tables

**Table 1:** General characteristic of participants.

	Nocturia (≥1 time)			Nocturia (≥2 times)		
	Control	Patient	P-value	Control	Patient	P-value
Number						
N	53,909	38,783		76,370	16,322	
%	58.2	41.8		82.4	17.6	
Age (years)	39.8	53.0	<0.001*	42.3	59.7	<0.001*
Walking days (d)	4.2	4.2	0.788	4.2	4.2	0.588
Married (%)			<0.001^†^			<0.001^†^
Yes	59.8	40.2		85.3	14.7	
No	83.1	16.9		95.9	4.1	
Education (%)			<0.001^†^			<0.001^†^
Low	38.4	61.6		69.1	30.9	
Middle	64.9	35.1		88.9	11.1	
High	75.2	24.8		93.7	6.3	
Occupation (%)			<0.001^†^			<0.001^†^
Manager, expert, specialist, clerk	73.2	26.8		93.7	6.3	
Service worker, salesperson	72.1	27.9		93.3	6.7	
Technician, mechanic, production worker, engineer	68.4	31.6		92.1	7.9	
Farmer, fisher, labourer, soldier	53.9	46.1		81.4	18.6	
Unemployed, student	56.7	43.3		77.2	22.8	
Income (%)			<0.001^†^			<0.001^†^
Lowest	46.8	53.2		70.7	29.3	
Low-middle	62.3	37.7		86.0	14.0	
Upper-middle	70.2	29.8		91.7	8.3	
Highest	71.3	28.7		92.6	7.4	
BMI (%)			<0.001^†^			<0.001^†^
<18.5 kg/m^2^	56.6	43.4		74.9	25.1	
≥18.5, <25 kg/m^2^	65.6	34.4		87.9	12.1	
≥25 kg/m^2^	67.1	32.9		89.6	10.4	
Smoking (%)			<0.001^†^			<0.001^†^
None	69.4	30.6		89.9	10.1	
Past smoker	53.0	47.0		81.0	19.0	
Current smoker	71.8	28.2		91.4	8.6	
Alcohol (%)			<0.001^†^			<0.001^†^
None	54.5	45.5		78.0	22.0	
≤1 time a month	68.4	31.6		89.5	10.5	
2–4 times a month	71.7	28.3		92.1	7.9	
≥2 times a week	64.5	35.5		88.3	11.7	
Sleep (%)			<0.001^†^			<0.001^†^
≤6 h	66.9	33.1		88.8	11.2	
7–8 h	65.6	34.4		88.2	11.8	
≥9 h	51.7	48.3		74.1	25.9	
Stress (%)			<0.001^†^			<0.001^†^
No	58.3	41.7		82.2	17.8	
Some	67.5	32.5		89.7	10.3	
Moderate	67.3	32.7		88.8	11.2	
Severe	65.6	34.4		86.6	13.4	
Hypertension (%)			<0.001^†^			<0.001^†^
Yes	45.4	54.6		74.5	25.5	
No	70.0	30.0		90.8	9.2	
Diabetes mellitus (%)			<0.001^†^			<0.001^†^
Yes	39.3	60.7		70.8	29.2	
No	67.7	32.3		89.3	10.7	
Hyperlipidaemia (%)			<0.001^†^			<0.001^†^
Yes	51.2	48.8		81.5	18.5	
No	67.3	32.7		88.8	11.2	
Cerebral stroke (%)			<0.001^†^			<0.001^†^
Yes	24.0	76.0		54.2	45.8	
No	66.4	33.6		88.5	11.5	

^*^Linear regression analysis with complex sampling; Significant at P < 0.05. ^†^Chi-square test with Rao-Scott correction; Significant at P < 0.05.

**Table 2:** Adjusted odd ratios of possible risk factors for nocturia (≥1 time a night; ≥2 times a night) using multiple logistic regression analysis with complex sampling.

	≥1 time a night			≥2 times a night		
	AOR	95% CI	P-value	AOR	95% CI	P-value
Age (10-year interval)	1.44	1.39–1.50	<0.001*	1.90	1.84–1.96	<0.001*
Walking days	0.99	0.98–1.00	0.376	1.00	0.99–1.00	0.264
Married			0.054			<0.001*
Yes	0.93	0.87–1.00		0.72	0.64–0.82	
No	1			1		
Education			<0.001*			<0.001*
Low	1.27	1.20–1.36		1.34	1.23–1.45	
Middle	1.04	0.99–1.09		1.07	0.99–1.16	
High	1			1		
Occupation			<0.001*			<0.001*
Manager, expert, specialist, clerk	1			1		
Service worker, salesperson	1.01	0.94–1.08		0.92	0.82–1.03	
Technician, mechanic, production worker, engineer	1.04	0.98–1.11		0.96	0.87–1.07	
Farmer, fisher, labourer, soldier	1.18	1.11–1.26		1.21	1.10–1.33	
Unemployed, student	1.21	1.13–1.29		1.44	1.31–1.58	
Income			<0.001*			<0.001*
Lowest	1.27	1.19–1.36		1.50	1.38–1.63	
Low-middle	1.13	1.07–1.19		1.20	1.10–1.30	
Upper-middle	1.00	0.95–1.06		0.99	0.91–1.07	
Highest	1			1		
BMI			0.022*			<0.001*
<18.5 kg/m^2^	1.19	1.05–1.34		1.54	1.34–1.77	
≥18.5, <25 kg/m^2^	1			1		
≥25 kg/m^2^	1.00	0.96–1.05		0.99	0.94–1.06	
Smoking			<0.001*			<0.001*
None	1			1		
Past smoker	1.05	1.00–1.11		1.04	0.97–1.12	
Current smoker	0.76	0.72–0.80		0.80	0.72–0.86	
Alcohol			<0.001*			0.063
None	1			1		
≤1 time a month	1.08	1.01–1.15		0.95	0.87–1.04	
2–4 times a month	1.10	1.03–1.16		0.93	0.86–1.00	
≥2 times a week	1.15	1.09–1.21		1.02	0.95–1.09	
Sleep			<0.001*			<0.001*
≤6 h	0.91	0.88–0.95		0.93	0.88–0.98	
7–8 h	1			1		
≥9 h	1.33	1.18–1.50		1.46	1.29–1.65	
Stress			<0.001*			<0.001*
No	1			1		
Some	1.11	1.05–1.16		1.07	1.01–1.15	
Moderate	1.23	1.16–1.31		1.37	1.27–1.48	
Severe	1.38	1.23–1.55		1.69	1.46–1.97	
Hypertension			0.001*			<0.001*
Yes	1.09	1.03–1.15		1.17	1.10–1.24	
No	1			1		
Diabetes mellitus			<0.001*			<0.001*
Yes	1.32	1.23–1.41		1.30	1.21–1.40	
No	1			1		
Hyperlipidaemia			<0.001*			<0.001*
Yes	1.28	1.20–1.36		1.17	1.08–1.27	
No	1			1		
Cerebral Stroke			<0.001*			<0.001*
Yes	1.63	1.40–1.89		1.44	1.25–1.65	
No	1			1		

^*^Significant at P < 0.05.
