# Supplementary material for: Analysis of the Prevalence and Factors Associated with Nocturia in Adult Korean Men
Source: Sci Rep. 2017 Jan 31;7:41714. doi: 10.1038/srep41714 (PMC5282484; doi:10.1038/srep41714)
Supplement: Supplementary Table S1 [file srep41714-s1.doc]

**Analysis of the Prevalence and Factors Associated with Nocturia in Adult Korean Men**

So Young Kim, MD^1+^, Woojin Bang, MD^2+^, Min-Su Kim, MD^3^, Bumjung Park, MD^4^, Jin-Hwan Kim, MD^5^, Hyo Geun Choi, MD^4^

^1^Department of Otorhinolaryngology-Head & Neck Surgery and Cancer Research Institute, Seoul National University College of Medicine, Seoul, Korea

^2^Department of Urology, Hallym University Sacred Heart Hospital, Hallym University Sacred Heart Hospital, Anyang, Korea

^3^Department of Otorhinolaryngology-Head & Neck Surgery, Korea University Ansan Hospital, Ansan, Korea

^4^Department of Otorhinolaryngology-Head & Neck Surgery, Hallym University College of Medicine, Anyang, Korea

^5^Department of Otorhinolaryngology-Head & Neck Surgery, Hallym University College of Medicine, Seoul, Korea

* Correspondence: Hyo Geun Choi, Email: [pupen@naver.com](mailto:pupen@naver.com)

+These authors contributed equally to this study

Supplementary Table S1 Odd ratios of possible risk factors for nocturia (≥ 1 time a night; ≥ 2 times a night) using simple logistic regression analysis with complex sampling

|  | |  | ≥ 1 time a night | | | ≥ 2 times a night | | |
| --- | --- | --- | --- | --- | --- | --- | --- | --- |
|  | |  | OR | 95% CI | P-value | OR | 95% CI | P-value |
| Age (10 years) | | | 1.89 | 1.86-1.91 | <0.001* | 2.23 | 2.28-2.28 | <0.001* |
| Walking Day | | | 1.00 | 1.00-1.01 | 0.788 | 1.00 | 0.99-1.01 | 0.588 |
| Marriage | | |  |  | <0.001* |  |  | <0.001* |
|  | Yes | | 2.69 | 2.35-3.09 |  | 4.06 | 3.70-4.44 |  |
|  | No | | 1 |  |  | 1 |  |  |
| Education | | |  |  | <0.001* |  |  | <0.001* |
|  | Low | | 4.86 | 4.65-5.08 |  | 6.59 | 6.20-6.99 |  |
|  | Middle | | 1.64 | 1.58-1.71 |  | 1.85 | 1.74-1.97 |  |
|  | High | | 1 |  |  | 1 |  |  |
| Occupation | | |  |  | <0.001* |  |  | <0.001* |
|  | Manager, Expert, Specialist, Clerk | | 1 |  |  | 1 |  |  |
|  | Service worker, salesperson | | 1.06 | 1.00-1.13 |  | 1.08 | 0.97-1.20 |  |
|  | Technician, Mechanics, Production worker, Engineer | | 1.26 | 1.20-1.33 |  | 1.28 | 1.17-1.40 |  |
|  | Farmer, Fisher, Laborer, Soldier | | 2.24 | 2.22-2.46 |  | 3.43 | 3.17-3.70 |  |
|  | Unemployed, Student | | 2.09 | 1.99-2.20 |  | 4.42 | 4.10-4.76 |  |
| Income | | |  |  | <0.001* |  |  | <0.001* |
|  | Lowest | | 2.83 | 2.68-2.98 |  | 5.15 | 4.81-5.52 |  |
|  | Low-middle | | 1.50 | 1.44-1.57 |  | 2.03 | 1.89-2.18 |  |
|  | Upper-middle | | 1.06 | 1.01-1.11 |  | 1.12 | 1.04-1.21 |  |
|  | Highest | | 1 |  |  | 1 |  |  |
| BMI | | |  |  | <0.001* |  |  | <0.001* |
|  | <18.5 kg/m^2^ | | 1.46 | 1.32-1.62 |  | 2.43 | 2.17-2.72 |  |
|  | ≥18.5, <25 kg/m^2^ | | 1 |  |  | 1 |  |  |
|  | ≥25 kg/m^2^ | | 0.94 | 0.90-0.97 |  | 0.84 | 0.79-0.88 |  |
| Smoking | | |  |  | <0.001* |  |  | <0.001* |
|  | None | | 1 |  |  | 1 |  |  |
|  | Past smoker | | 2.00 | 1.92-2.10 |  | 2.09 | 1.97-2.23 |  |
|  | Current smoker | | 0.89 | 0.85-0.93 |  | 0.84 | 0.78-0.89 |  |
| Alcohol | | |  |  | <0.001* |  |  | <0.001* |
|  | None | | 1 |  |  | 1 |  |  |
|  | ≤ 1 time a month | | 0.55 | 0.52-0.58 |  | 0.42 | 0.39-0.45 |  |
|  | 2-4 times a month | | 0.47 | 0.45-0.50 |  | 0.30 | 0.28-0.33 |  |
|  | ≥ 2 times a week | | 0.66 | 0.63-0.69 |  | 0.47 | 0.44-0.50 |  |
| Sleep | | |  |  | <0.001* |  |  | <0.001* |
|  | ≤ 6h | | 0.94 | 0.91-0.97 |  | 0.95 | 0.90-0.99 |  |
|  | 7-8h | | 1 |  |  | 1 |  |  |
|  | ≥ 9h | | 1.78 | 1.62-1.96 |  | 2.62 | 2.37-2.91 |  |
| Stress | | |  |  | <0.001* |  |  | <0.001* |
|  | No | | 1 |  |  | 1 |  |  |
|  | Some | | 0.67 | 0.64-0.70 |  | 0.53 | 0.50-0.56 |  |
|  | Moderate | | 0.68 | 0.64-0.71 |  | 0.59 | 0.55-0.63 |  |
|  | Severe | | 0.73 | 0.66-0.81 |  | 0.71 | 0.63-0.81 |  |
| Hypertension | | |  |  | <0.001* |  |  | <0.001* |
|  | Yes | | 2.80 | 2.69-2.92 |  | 3.39 | 3.23-3.56 |  |
|  | No | | 1 |  |  | 1 |  |  |
| Diabetes mellitus | | |  |  | <0.001* |  |  | <0.001* |
|  | Yes | | 3.24 | 3.05-3.44 |  | 3.45 | 3.24-3.68 |  |
|  | No | | 1 |  |  | 1 |  |  |
| Hyperlipidemia | | |  |  | <0.001* |  |  | <0.001* |
|  | Yes | | 1.97 | 1.86-2.08 |  | 1.79 | 1.67-1.92 |  |
|  | No | | 1 |  |  | 1 |  |  |
| Cerebral Stroke | | |  |  | <0.001* |  |  | <0.001* |
|  | Yes | | 6.26 | 5.43-7.22 |  | 6.54 | 5.77-7.41 |  |
|  | No | | 1 |  |  | 1 |  |  |

* Significance at P < 0.05
