# Supplementary material for: Analysis of the Prevalence and Factors Associated with Nocturia in Adult Korean Men
Source: Sci Rep. 2017 Jan 31;7:41714. doi: 10.1038/srep41714 (PMC5282484; doi:10.1038/srep41714)
Supplement: Supplementary Notes S2 [file srep41714-s2.doc]

**Analysis of the Prevalence and Factors Associated with Nocturia in Adult Korean Men**

So Young Kim, MD^1+^, Woojin Bang, MD^2+^, Min-Su Kim, MD^3^, Bumjung Park, MD^4^, Jin-Hwan Kim, MD^5^, Hyo Geun Choi, MD^4^

^1^Department of Otorhinolaryngology-Head & Neck Surgery and Cancer Research Institute, Seoul National University College of Medicine, Seoul, Korea

^2^Department of Urology, Hallym University Sacred Heart Hospital, Hallym University Sacred Heart Hospital, Anyang, Korea

^3^Department of Otorhinolaryngology-Head & Neck Surgery, Korea University Ansan Hospital, Ansan, Korea

^4^Department of Otorhinolaryngology-Head & Neck Surgery, Hallym University College of Medicine, Anyang, Korea

^5^Department of Otorhinolaryngology-Head & Neck Surgery, Hallym University College of Medicine, Seoul, Korea

* Correspondence: Hyo Geun Choi, Email: [pupen@naver.com](mailto:pupen@naver.com)

+These authors contributed equally to this study

**Supplementary Notes S2. The definitions of nocturia compared in the present study**

The frequency-volume chart is a gold-standard method to survey nocturia. However, many studies using questionnaires such as “How many times did you typically get up at night to urinate in the past month?” in the present study. This study cited the researches using questionnaires to discuss on the result of the present study. The examples of researches adopted similar questionnaires with the present study to define nocturia as follows.

Tikkinen, K. A., Tammela, T. L., Huhtala, H. & Auvinen, A. Is nocturia equally common among men and women? A population based study in Finland. *The Journal of urology* **175**, 596-600, doi:10.1016/S0022-5347(05)00245-4 (2006).

Madhu, C. *et al.* Nocturia: risk factors and associated comorbidities; findings from the EpiLUTS study. *International journal of clinical practice* **69**, 1508-1516, doi:10.1111/ijcp.12727 (2015).

The

prevalence of lower urinary tract symptoms (LUTS)

in the USA, the UK and Sweden: results from the

Epidemiology of LUTS (EpiLUTS) study

Fitzgerald, M. P., Litman, H. J., Link, C. L., McKinlay, J. B. & Bach Survey Investigators. The association of nocturia with cardiac disease, diabetes, body mass index, age and diuretic use: results from the BACH survey. *The Journal of urology* **177**, 1385-1389, doi:10.1016/j.juro.2006.11.057 (2007).

Negoro, H. *et al.* Underweight body mass index is a risk factor of mortality in outpatients with nocturia in Japan. *BMC research notes* **8**, 490, doi:10.1186/s13104-015-1456-6 (2015).

Afsar, B. & Elsurer, R. Central hemodynamics, vascular stiffness, and nocturia in patients with type 2 diabetes. *Renal failure* **37**, 359-365, doi:10.3109/0886022X.2015.1088335 (2015).
